# Computed Tomographic Features Associated with Pulmonary Melioidosis versus Pulmonary Tuberculosis in Diabetic Patients: A Retrospective Comparative Cohort Study

**DOI:** 10.4269/ajtmh.26-0337

**Published:** 2026-10-13

**Authors:** Nitin Gupta, Chiranjay Mukhopadhyay, Tirlangi Praveen Kumar, Kavita Salian, Priya P. S., Sundeep Malla, Rahul Magazine, Steven Van Den Broucke, Erika Vlieghe, Emmanuel Bottieau

**Affiliations:** ^1^ Department of Infectious Diseases, Kasturba Medical College, Manipal Academy of Higher Education, Manipal, India;; ^2^ Department of Clinical Sciences, Institute of Tropical Medicine, Antwerp, Belgium;; ^3^ University of Antwerp, Antwerp, Belgium;; ^4^ Department of Microbiology, Kasturba Medical College, Manipal Academy of Higher Education, Manipal, India;; ^5^ Department of Radiodiagnosis and Imaging, Kasturba Medical College, Manipal Academy of Higher Education, Manipal, India;; ^6^ Department of Radiodiagnosis, Amrita Institute of Medical Sciences, Faridabad, India;; ^7^ Department of Respiratory Medicine, Kasturba Medical College, Manipal Academy of Higher Education, Manipal, India;; ^8^ University Hospital Antwerp, Antwerp, Belgium

## Abstract

Pulmonary melioidosis is frequently mistaken for pulmonary tuberculosis because of overlapping clinical and radiological features, often delaying appropriate treatment. We conducted a retrospective comparative cohort study at a tertiary care center in southern India between January 2018 and August 2025 to compare chest computed tomography (CT) features of microbiologically confirmed melioidosis and tuberculosis in diabetic adults who underwent CT within 30 days of symptom onset. A total of 151 patients were included: 46 with melioidosis and 105 with tuberculosis. Cavitation (*n* = 81/105 [77.1%] versus *n* = 20/46 [43.5%], *P* <0.001) and hydropneumothorax (*n* = 11/105 [10.5%] versus *n* = 0/46 [0%], *P* = 0.019) were more common in tuberculosis. In contrast, when compared with tuberculosis, melioidosis was associated with more frequent bilateral lung involvement (*n* = 37/105 [35.2%] versus *n* = 26/46 [56.5%], *P* = 0.015), pleural effusion (*n* = 28/105 [26.7%] versus *n* = 23/46 [50.0%], *P* = 0.005), and hilar lymphadenopathy (*n* = 6/105 [5.7%] versus *n* = 8/46 [17.4%], *P* = 0.032). Despite these differences, there was substantial overlap in CT findings, with upper-lobe predominance, centrilobular nodules, and mediastinal lymphadenopathy seen in both groups. In multivariable analysis, cavitation was associated with significantly lower odds of melioidosis than tuberculosis (adjusted odds ratio: 0.25, 95% CI: 0.12–0.54, *P* <0.001). Pulmonary melioidosis and pulmonary tuberculosis, therefore, show substantial overlap on chest CT in diabetic patients, and imaging should be interpreted as an adjunct to targeted microbiological diagnosis rather than a substitute for it.

## INTRODUCTION

Melioidosis and tuberculosis (TB) are important causes of lung infection in tropical and subtropical settings, particularly among patients with diabetes mellitus (DM).^1^^,^^2^ Although *Mycobacterium tuberculosis* remains a well-established cause of respiratory infection worldwide, *Burkholderia pseudomallei* has emerged as an increasingly recognized pathogen in South and Southeast Asia.^3^ In endemic regions, the two diseases often overlap clinically and on imaging, creating significant diagnostic challenges.^4^^,^^5^

Definitive diagnosis of both conditions relies on microbiological confirmation and often requires invasive sampling when noninvasive respiratory specimens are unavailable or nondiagnostic. In routine practice, however, patients with radiological findings considered classical for TB are often started empirically on antitubercular therapy before a definitive microbiological diagnosis is established.^6^^,^^7^ This problem is not unique to melioidosis as a range of endemic infections, including nontuberculous mycobacterial disease, chronic fungal infections, and parasitic infections such as paragonimiasis, may also mimic TB clinically and radiologically. Some of these patients may, in fact, have melioidosis, particularly in regions where its prevalence is increasing and awareness remains limited. Although the radiological features of TB are well described, those of melioidosis remain less well characterized, and there is a need to define imaging features that may help distinguish the two conditions.^8^ We, therefore, conducted a retrospective comparative cohort study to compare chest computed tomography (CT) findings in diabetic adults with microbiologically confirmed melioidosis and TB to support earlier microbiological evaluation and more timely treatment in this high-risk population.

## MATERIALS AND METHODS

This retrospective comparative cohort study included adults ages 18 years old or older admitted to a tertiary care center in southern India between January 2018 and August 2025 (Institutional Ethics Committee approval no. IEC-1-253). This study was conducted and reported in accordance with the Strengthening the Reporting of Observational Studies in Epidemiology guidelines, and the completed checklist is provided in Supplemental Appendix 1.

Pulmonary melioidosis was defined by isolation of *B. pseudomallei* from blood, respiratory, or other sterile-site specimens in patients with compatible respiratory symptoms and imaging evidence of lung parenchymal disease. Pulmonary TB was defined by positive sputum smear microscopy, GeneXpert (Cepheid, Sunnyvale, CA, USA) *Mycobacterium tuberculosis*/rifampicin (MTB/RIF) assay, or mycobacterial culture in patients with compatible respiratory symptoms and imaging evidence of lung parenchymal disease. Patients with pulmonary TB or pulmonary melioidosis were first screened for the availability of chest CT, after which those with CT performed within 30 days of symptom onset and those with documented DM were included in the final analysis. To reduce potential selection and information bias, eligibility was restricted to diabetic patients who underwent CT within 30 days of symptom onset, and CT scans were reviewed in random order by an experienced radiologist blinded to the final diagnosis, with findings verified by a second radiologist. Diabetes mellitus was selected as the common host factor because it is a major risk factor for melioidosis and an established risk factor for pulmonary TB,^9^^,^^10^ and it may also modify the radiological presentation of TB.^11^^,^^12^ Restricting both groups to patients with DM was intended to reduce host-related heterogeneity and allow for a more focused comparison of CT findings attributable to the underlying infection.

Demographic and clinical variables, including age, sex, season of presentation (monsoon versus nonmonsoon), glycated hemoglobin (HbA1c), cough, hemoptysis, dyspnea, and extrapulmonary involvement, were extracted from medical records. Time from symptom onset to the first CT scan, time to diagnosis from hospital admission, and in-hospital mortality were also recorded. Radiological terms and definitions were based on the Fleischner Society glossary of thoracic imaging terms, with study-specific operational definitions provided separately in Table 1.^13^

**Table 1:** Study definitions of disease categories and radiological variables

Domain and Variable	Operational Definition
Disease definitions	
Pulmonary melioidosis	Isolation of *Burkholderia pseudomallei* from blood, respiratory, or other sterile-site specimens in a patient with compatible respiratory symptoms and imaging evidence of lung parenchymal disease
Pulmonary tuberculosis	Positive sputum smear microscopy, GeneXpert MTB/RIF assay, or culture for *Mycobacterium tuberculosis* in a patient with compatible respiratory symptoms and imaging evidence of lung parenchymal disease
Parenchymal findings	
Consolidation	Homogeneous increased pulmonary attenuation obscuring vessel and airway margins
Cavitation	Gas-filled space with a definable wall within a pulmonary nodule, mass, or area of consolidation
Thick-walled cavity	Cavity with maximum wall thickness greater than 4 mm on axial lung window images
Distribution	
Lobar predominance	Classified as upper-lobe predominance or lower-lobe predominance when more than 50% of the overall disease burden involved the respective lobar region; otherwise classified as no lobar predominance
Laterality	Classified as unilateral or bilateral when more than 50% of the overall disease burden involved one lung or both lungs, respectively
Nodules	
Pulmonary nodule	Rounded or irregular opacity measuring up to 30 mm in diameter
Centrilobular nodules	Nodules are distributed in the centrilobular region
Random nodules	Nodules are distributed randomly without a centrilobular pattern
Pleural findings	
Pleural effusion	Pleural fluid collection identified on CT
Hydropneumothorax	Presence of both pleural air and pleural fluid
Empyema	Pleural collection considered consistent with infected pleural fluid
Vascular and ancillary findings	
Septic emboli	Peripheral nodular or cavitary lesions considered compatible with embolic infection
Rasmussen aneurysm	Pulmonary artery pseudoaneurysm adjacent to a cavitary lesion
Lymph nodes	
Mediastinal lymphadenopathy (of any station)	Lymph node involving any station with a short-axis diameter greater than 10 mm
Hilar, pretracheal, paratracheal, subcarinal nodes	Enlargement (short-axis diameter >10 mm) of individual stations was recorded separately

CT = computed tomography; MTB = *Mycobacterium tuberculosis*; RIF = rifampicin. Radiological terms and definitions were based on the Fleischner Society glossary of thoracic imaging terms with study-specific operational definitions applied where needed.

All data were entered into a standardized electronic database and analyzed using SPSS v. 26 (IBM Corp., Armonk, NY, USA). Categorical variables were summarized as counts and percentages, and they were compared using the χ^2^ test or the Fisher exact test as appropriate. Continuous variables were summarized as means with SDs or medians with interquartile ranges depending on the distribution, and they were compared using the independent-samples *t*-test or the Mann–Whitney *U* test. Odds ratios with 95% CIs were calculated to assess the strength of association between individual CT features and pulmonary melioidosis. A two-sided *P*-value of less than 0.05 was considered statistically significant.

Logistic regression analysis was performed to identify chest CT features associated with pulmonary melioidosis, with melioidosis coded as the dependent outcome and pulmonary TB coded as the reference category. Imaging variables identified on the univariate analysis were entered into the multivariable model as binary covariates. A stepwise elimination approach was used to sequentially remove variables that did not contribute independently to the model, aiming to derive a parsimonious, clinically interpretable final model. Collinearity between candidate variables was assessed before model reduction. Adjusted odds ratios with corresponding 95% CIs were calculated to quantify the strength of association between retained CT features and pulmonary melioidosis. There were no missing data for variables included in the primary regression model. No prespecified subgroup, interaction, or sensitivity analyses were performed.

## RESULTS

During the study period, 105 patients with pulmonary TB and 46 patients with pulmonary melioidosis who had documented DM and chest CT performed within 30 days of symptom onset were included in the final analysis (Supplemental Figure 1). Mean age (55.2 ± 12.7 versus 53.5 ± 12.6 years old, *P* = 0.434), male sex (*n* = 82/105 [78.1%] versus *n* = 40/46 [87.0%], *P* = 0.203), and HbA1c (9.56 ± 2.64 versus 10.21 ± 2.55, *P* = 0.159) did not differ significantly between the groups. Presentation during the monsoon months was more common in melioidosis (*n* = 31/105 [29.5%] versus *n* = 30/46 [65.2%], *P* <0.001). Cough was more frequent in TB (*n* = 103/105 [98.1%] versus *n* = 34/46 [73.9%], *P* <0.001), whereas dyspnea (*n* = 31/105 [29.5%] versus *n* = 22/46 [47.8%], *P* = 0.041) and extrapulmonary abscesses (*n* = 3/105 [2.9%] versus *n* = 23/46 [50.0%], *P* <0.001) were more common in melioidosis. Hemoptysis (*n* = 13/105 [12.4%] versus *n* = 3/46 [6.5%], *P* = 0.393), duration of illness before CT (13.1 ± 10.7 versus 12.5 ± 9.8 days, *P* = 0.749), and in-hospital mortality (*n* = 3/105 [2.9%] versus *n* = 2/46 [4.3%], *P* = 0.641) did not differ significantly. Time to diagnosis from admission was longer in melioidosis (5.6 ± 4.4 versus 8.7 ± 5.1 days, *P* <0.001) (Tables 2 and 3).

**Table 2:** Baseline demographic and clinical characteristics of patients with pulmonary tuberculosis and pulmonary melioidosis

Variable	Tuberculosis (*n* = 105)	Melioidosis (*n* = 46)	*P*-Value
Age, years, mean ± SD	55.2 ± 12.7	53.5 ± 12.6	0.434
Male sex, *n* (%)	82 (78.1)	40 (87.0)	0.203
HbA1c, mean ± SD	9.56 ± 2.64	10.21 ± 2.55	0.159
Monsoon, June to September, *n* (%)	31 (29.5)	30 (65.2)	<0.001
Cough, *n* (%)	103 (98.1)	34 (73.9)	<0.001
Hemoptysis, *n* (%)	13 (12.4)	3 (6.5)	0.393
Dyspnea, *n* (%)	31 (29.5)	22 (47.8)	0.041
Extrapulmonary abscesses, *n* (%)	3 (2.9)	23 (50.0)	<0.001
Duration of illness before CT, days, mean ± SD	13.1 ± 10.7	12.5 ± 9.8	0.749
Time to diagnosis from admission, days, mean ± SD	5.6 ± 4.4	8.7 ± 5.1	<0.001
In-hospital mortality, *n* (%)	3 (2.9)	2 (4.3)	0.641

CT = computed tomography; HbA1c = glycated hemoglobin.

**Table 3:** Chest computed tomography features comparing pulmonary tuberculosis and pulmonary melioidosis in diabetic patients

Domain and Feature	Tuberculosis	Melioidosis	*P*-Value
Parenchymal findings			
Consolidation	105/105 (100)	46/46 (100)	NA
Cavitation (any)	81/105 (77.1)	20/46 (43.5)	<0.001
Lobar predominance			
Upper-lobe predominance	43/105 (41.0)	22/46 (47.8)	0.478
Lower-lobe predominance	24/105 (22.9)	12/46 (26.1)	
No lobar predominance	38/105 (36.2)	12/46 (26.1)	
Laterality			
Unilateral lung involvement	68/105 (64.8)	20/46 (43.5)	0.015
Bilateral lung involvement	37/105 (35.2)	26/46 (56.5)	
Nodule distribution			
Centrilobular nodules	81/105 (77.1)	31/46 (67.4)	0.208
Random nodules	2/105 (1.9)	3/46 (6.5)	0.166
Pleural disease			
Pleural effusion	28/105 (26.7)	23/46 (50.0)	0.005
Hydropneumothorax	11/105 (10.5)	0/46 (0)	0.019
Empyema	4/105 (3.8)	1/46 (2.2)	1.000
Vascular/ancillary signs			
Septic emboli	0/105 (0)	2/46 (4.3)	0.091
Rasmussen aneurysm	3/105 (2.9)	0/46 (0)	0.553
Lymph nodes			
Any mediastinal lymphadenopathy	49/105 (46.7)	19/46 (41.3)	0.542
Hilar nodes	6/105 (5.7)	8/46 (17.4)	0.032
Pretracheal nodes	14/105 (13.3)	3/46 (6.5)	0.274
Paratracheal nodes	44/105 (41.9)	16/46 (34.8)	0.472
Subcarinal nodes	25/105 (23.8)	8/46 (17.4)	0.521

NA = not applicable. Lobar predominance was classified as upper-lobe predominance, lower-lobe predominance, or no lobar predominance based on the overall distribution of disease. Laterality was classified as unilateral or bilateral lung involvement. A thick-walled cavity was defined as a cavity with a maximum wall thickness greater than 4 mm. Mediastinal lymphadenopathy was defined as mediastinal lymph nodes with a short-axis diameter greater than 10 mm.

Chest CT findings showed substantial overlap between the two diseases. Consolidation was present in all patients (*n* = 105/105 [100%] versus *n* = 46/46 [100%]). Cavitation was more common in TB (*n* = 81/105 [77.1%] versus *n* = 20/46 [43.5%], *P* <0.001), although it was present in both groups; except for one patient with TB, all cavities were thick walled. Upper-lobe predominance (*n* = 43/105 [41.0%] versus *n* = 22/46 [47.8%], *P* = 0.478), centrilobular nodules (*n* = 81/105 [77.1%] versus *n* = 31/46 [67.4%], *P* = 0.208), random nodules (*n* = 2/105 [1.9%] versus *n* = 3/46 [6.5%], *P* = 0.166), any mediastinal lymphadenopathy (*n* = 49/105 [46.7%] versus *n* = 19/46 [41.3%], *P* = 0.542), Rasmussen aneurysm (*n* = 3/105 [2.9%] versus *n* = 0/46 [0%], *P* = 0.553), and empyema (*n* = 4/105 [3.8%] versus *n* = 1/46 [2.2%], *P* = 1.000) did not differ significantly between groups. In contrast, bilateral lung involvement (*n* = 37/105 [35.2%] versus *n* = 26/46 [56.5%], *P* = 0.015), pleural effusion (*n* = 28/105 [26.7%] versus *n* = 23/46 [50.0%], *P* = 0.005), and hilar lymphadenopathy (*n* = 6/105 [5.7%] versus *n* = 8/46 [17.4%], *P* = 0.032) were more common in melioidosis, whereas hydropneumothorax was more common in TB (*n* = 11/105 [10.5%] versus *n* = 0/46 [0%], *P* = 0.019). Septic emboli were seen only in melioidosis (*n* = 0/105 [0%] versus *n* = 2/46 [4.3%], *P* = 0.091), but this difference was not statistically significant. Pretracheal (*n* = 14/105 [13.3%] versus *n* = 3/46 [6.5%], *P* = 0.274), paratracheal (*n* = 44/105 [41.9%] versus *n* = 16/46 [34.8%], *P* = 0.472), and subcarinal lymphadenopathy (*n* = 25/105 [23.8%] versus *n* = 8/46 [17.4%], *P* = 0.521) also did not differ significantly.

In the multivariable logistic regression model including bilateral lung involvement, pleural effusion, hilar lymphadenopathy, and cavitation, only cavitation remained independently associated with lower odds of melioidosis (adjusted odds ratio: 0.25, 95% CI: 0.12–0.54, *P* <0.001) (Table 4). Bilateral lung involvement (adjusted odds ratio: 1.86, 95% CI: 0.86–4.02, *P* = 0.113), pleural effusion (adjusted odds ratio: 2.07, 95% CI: 0.94–4.56, *P* = 0.071), and hilar lymphadenopathy (adjusted odds ratio: 2.78, 95% CI: 0.81–9.49, *P* = 0.104) were not independently associated with melioidosis. The model showed reasonable performance (omnibus χ^2^ = 26.92, *P* <0.001; Hosmer–Lemeshow χ^2^ = 2.74, *P* = 0.841; overall classification accuracy: 77.5%) (Figures 1 and 2).

**Table 4:** Multivariable logistic regression model of computed tomography predictors of pulmonary melioidosis

CT Feature	Crude OR (95% CI)	aOR	95% CI for aOR	*P*-Value
Bilateral lung involvement	2.39 (1.18–4.85)	1.86	0.86–4.02	0.113
Cavitation	0.23 (0.11–0.48)	0.25	0.12–0.54	<0.001
Pleural effusion	2.75 (1.34–5.66)	2.07	0.94–4.56	0.071
Hilar lymphadenopathy	3.47 (1.13–10.67)	2.78	0.81–9.49	0.104

aOR = adjusted odds ratio; CT = computed tomography; OR = odds ratio. Melioidosis was coded as the dependent outcome with pulmonary tuberculosis as the reference category. Crude ORs were derived from univariable analysis, and aORs were obtained from the multivariable logistic regression model. Values greater than one favor pulmonary melioidosis, whereas values less than one favor pulmonary tuberculosis.

**Figure 1.**
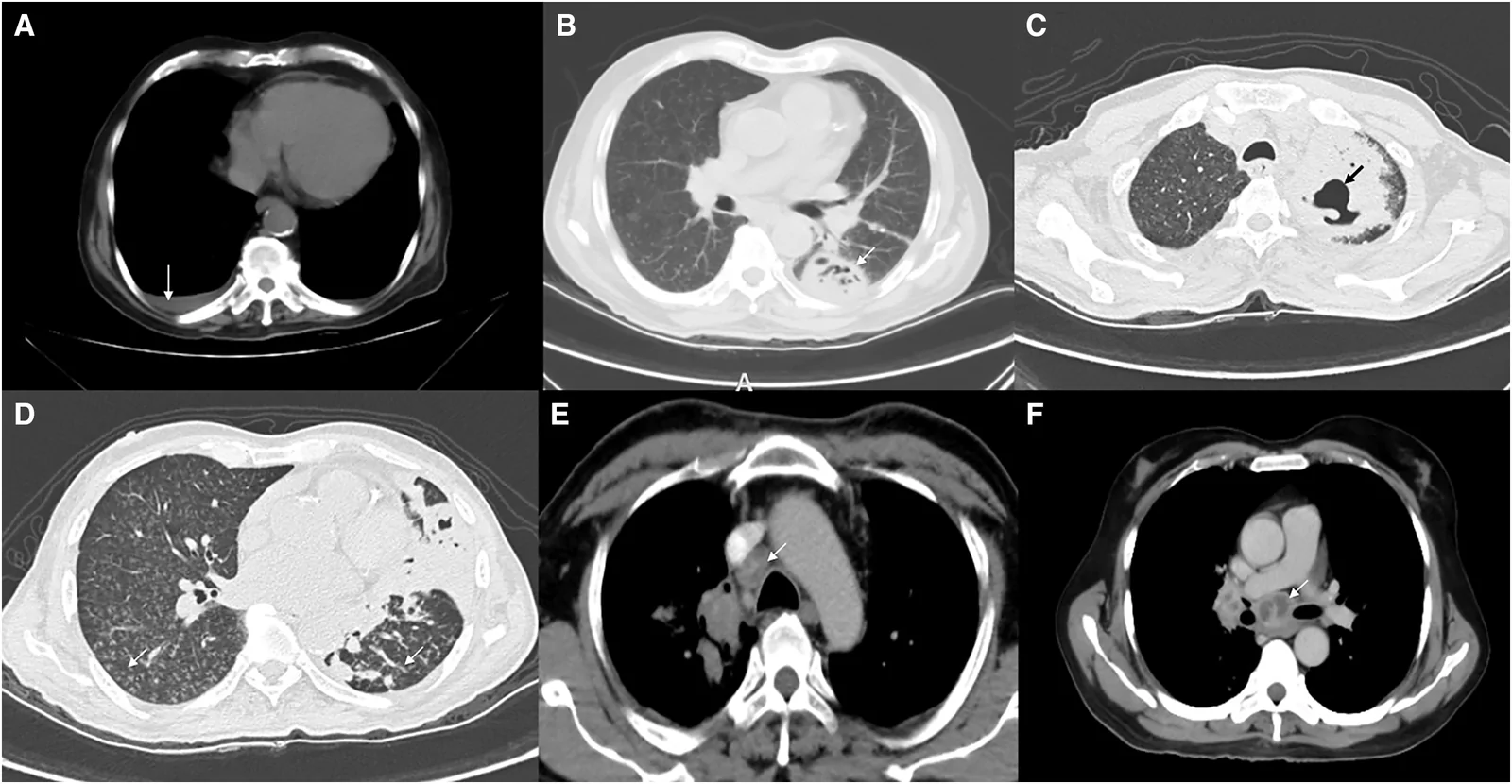
Computed tomography (CT) chest findings in a patient with pulmonary melioidosis. (**A**) Noncontrast axial CT chest image (mediastinal window) demonstrating mild right-sided pleural effusion. (**B**) Axial CT lung window image showing a large area of consolidation in the left lower lobe with central clearing. (**C**) Axial CT lung window image demonstrating a large, thick-walled cavity in the left upper lobe. (**D**) Axial CT lung window image showing multiple bilateral centrilobular nodules with a tree-in-bud appearance, consistent with endobronchial spread of infection. (**E**) Contrast-enhanced axial CT chest image (mediastinal window) demonstrating an enlarged necrotic right paratracheal lymph node. (**F**) Contrast-enhanced axial CT chest image (mediastinal window) demonstrating enlarged necrotic subcarinal and right hilar lymph nodes.

**Figure 2.**
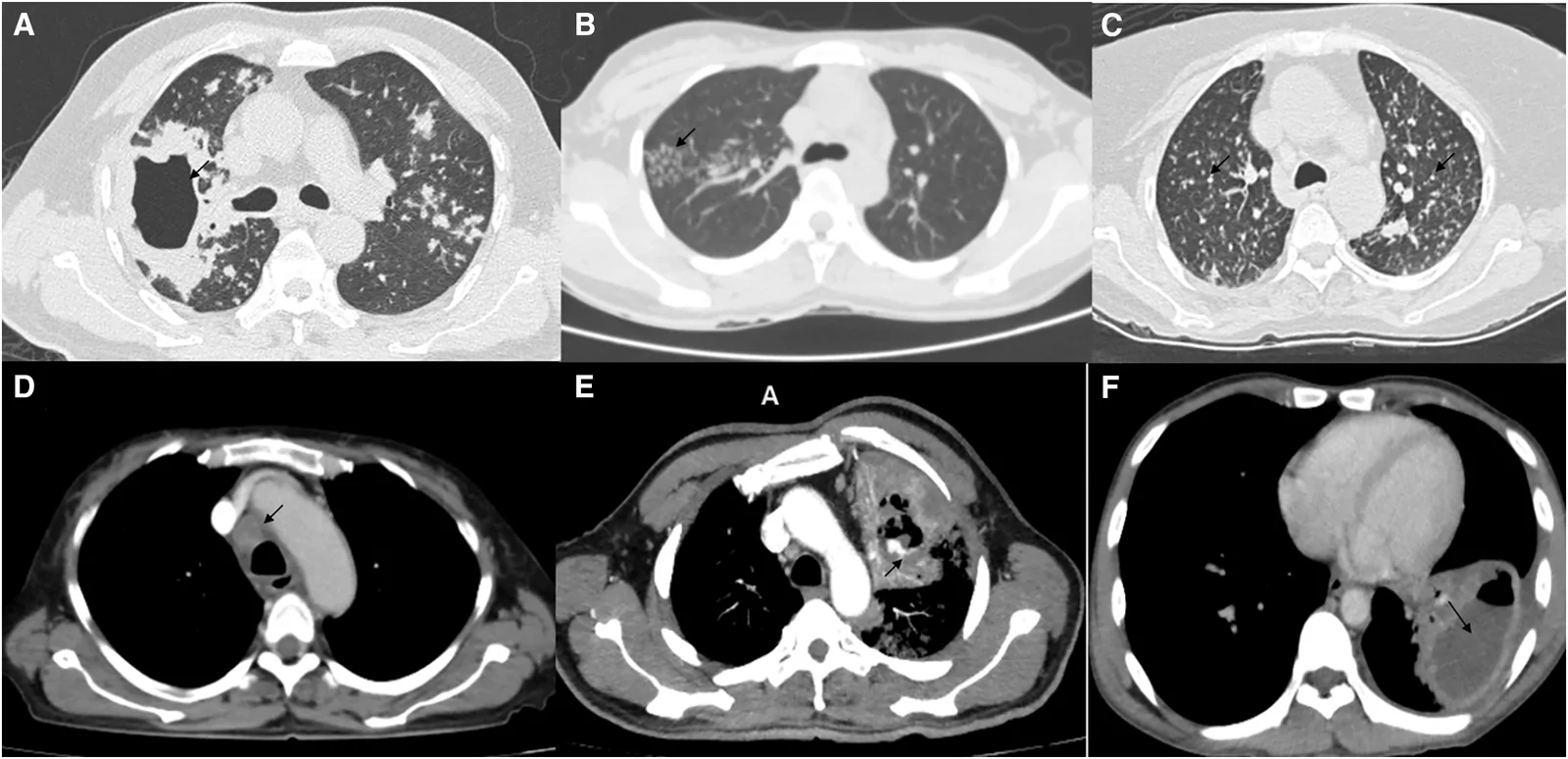
Computed tomography (CT) chest findings in a patient with pulmonary tuberculosis. (**A**) Axial CT lung window image demonstrating a large, thick-walled cavity in the right upper lobe. (**B**) Axial CT lung window image showing multiple centrilobular nodules in the right upper lobe with a tree-in-bud appearance, consistent with endobronchial spread of infection. (**C**) Axial CT lung window image demonstrating multiple randomly distributed nodules in both lung fields, suggestive of hematogenous dissemination. (**D**) Contrast-enhanced axial CT chest image (mediastinal window) demonstrating an enlarged necrotic right paratracheal lymph node. (**E**) Contrast-enhanced axial CT chest image demonstrating a Rasmussen aneurysm arising from the right upper lobar pulmonary artery adjacent to the cavitary lesion. (**F**) Contrast-enhanced axial CT chest image demonstrating a loculated empyema within the left pleural cavity.

## DISCUSSION

In this retrospective comparison of diabetic patients with pulmonary melioidosis and pulmonary TB, chest CT demonstrated substantial radiological overlap between the two diseases, with many findings occurring in both groups. Upper-lobe predominance, centrilobular nodules, and mediastinal lymphadenopathy were seen in both conditions, and even features that differed in frequency showed considerable overlap. Tuberculosis was more often associated with cavitation and hydropneumothorax, whereas melioidosis more commonly showed bilateral lung involvement, pleural effusion, and hilar lymphadenopathy. Overall, these findings suggest that although certain CT patterns may modestly shift diagnostic suspicion, the predominant message is one of overlap rather than clear radiological separation between pulmonary melioidosis and pulmonary TB.

Baseline comparability in host factors between the two groups was an important strength of the study. All included patients had DM, and glycemic control was similar between the groups, reducing the likelihood that differences in DM severity materially influenced the observed imaging patterns. In keeping with the known epidemiology of melioidosis, cases were also more frequent during the monsoon months.^3^ We could not reliably account for other recognized risk factors for melioidosis, such as soil exposure and alcohol consumption, because these are often incompletely captured in routine retrospective records, particularly among patients with TB.^14^ To minimize additional variation related to disease duration, we also restricted inclusion to patients who underwent chest CT within 30 days of symptom onset; accordingly, duration of illness at the time of CT did not differ significantly between the groups.

Clinical differences were also observed between the two groups. Cough was more common in TB, whereas dyspnea and extrapulmonary abscesses were more frequent in melioidosis. The higher frequency of extrapulmonary abscesses in melioidosis is biologically plausible as melioidosis is commonly bacteremic and often disseminates beyond the lungs to organs such as the liver, spleen, prostate, bone, joints, and parotid gland.^3^^,^^14^^,^^15^ Time to diagnosis from hospital admission was also slightly longer in melioidosis, likely reflecting lower initial clinical suspicion, greater diagnostic uncertainty in TB-endemic settings, and reliance on culture-dependent diagnostic techniques in contrast to the more established use of microscopy and molecular tests in TB.^2^ However, these clinical distinctions are already well recognized, and the purpose of the present study was not to re-establish known clinicobiological differences between the two infections. Instead, we specifically focused on chest CT findings to determine whether imaging alone could help differentiate pulmonary melioidosis from pulmonary TB in diabetic patients.

An important observation from this study is the degree of radiological overlap between pulmonary melioidosis and TB. Upper-lobe predominance, centrilobular nodules, and mediastinal lymphadenopathy were seen in both groups, and no single CT feature was completely disease specific. Although these findings are often regarded as classical for pulmonary TB, previous small case series of thoracic melioidosis have also highlighted upper-lobe infiltrates and centrilobular nodules.^16^^–^^18^ Even mediastinal lymph nodes, which are classically considered rare in melioidosis, were frequently observed in our study.^16^ This overlap is clinically important in TB-endemic regions.^8^ Our findings reinforce that melioidosis should remain an important differential diagnosis in diabetic patients with pulmonary infection, even when the CT appearance appears compatible with TB.

Although cavitation was significantly more frequent in pulmonary TB than in pulmonary melioidosis in our cohort, it was observed in both diseases. A cavity reflects parenchymal necrosis with evacuation of necrotic material through the bronchial tree, a process classically associated with pulmonary TB because of caseating necrosis and progressive tissue destruction.^19^ It is also possible that the stronger association of cavitation with TB in our cohort partly reflects the radiological phenotype of diabetic TB rather than TB more broadly.^12^ By contrast, the lower frequency of cavitation in melioidosis may relate to differences in disease evolution, with pulmonary melioidosis perhaps more often presenting as acute or multifocal consolidation rather than progressing to frank cavitary breakdown. However, this remains speculative and may also represent a chance finding in our dataset. We did not observe a meaningful difference between the two groups in terms of thick versus thin-walled cavities, and most cavities in both conditions were thick walled. Earlier descriptive literature has suggested that melioidosis cavities are often thin walled.^18^ Still, those observations were based on small radiological series and lacked a clear quantitative definition of cavity wall thickness.

Some imaging findings were almost exclusive to one disease and may reflect important differences in pathobiology. Hydropneumothorax and Rasmussen aneurysm were observed only in pulmonary TB in our cohort, whereas septic emboli were observed only in melioidosis, although the numbers were small. The occurrence of hydropneumothorax in TB likely reflects rupture of cavitary or necrotic pleuropulmonary disease into the pleural space with or without associated bronchopleural communication.^20^ Rasmussen aneurysm is also a classical complication of cavitary TB. It arises from weakening of the adjacent pulmonary arterial wall by chronic inflammatory destruction, making it biologically more consistent with long-standing necrotizing cavitary disease than with melioidosis.^21^ In contrast, septic emboli were identified only in melioidosis, which is plausible given melioidosis’s well-recognized tendency to be bacteremic and disseminated, with hematogenous seeding of the lungs and other organs.^22^ Although these findings are too infrequent to serve as reliable stand-alone discriminators, when present, they may provide useful diagnostic clues and further emphasize the predominantly cavitary-destructive pattern of TB as opposed to the more bacteremic and disseminated profile of melioidosis.

In resource-limited, TB-endemic settings, patients with pulmonary melioidosis may be easily misdiagnosed as having pulmonary TB, particularly when they present with chronic respiratory symptoms and CT findings, such as upper-lobe involvement, nodules, or cavitary disease. In such contexts, there is often a tendency to overdiagnose TB, and empirical antitubercular therapy may be started before a definitive microbiological diagnosis is established. This can delay appropriate treatment of melioidosis with potentially serious consequences. Our findings, therefore, have implications beyond radiological differentiation alone. They underscore the need to strengthen microbiology laboratory capacity for timely diagnosis of melioidosis and to improve clinical and radiological awareness of this infection in endemic regions. Training clinicians and radiologists to recognize melioidosis as an important differential diagnosis, especially in diabetic patients with bilateral disease, pleural effusion, or extrapulmonary abscesses, may help reduce diagnostic anchoring toward TB and support earlier initiation of appropriate investigations and therapy.

This study has several limitations. Its retrospective design introduces the possibility of selection and information bias. Chest CT was available only for patients who underwent scanning as part of routine clinical care, which may have preferentially included individuals who were clinically stable enough to undergo imaging and therefore, underrepresented the full spectrum of severe disease. The study was conducted at a single center and restricted to diabetic patients, which improved internal comparability but may limit generalizability to other populations, particularly nondiabetic patients. The stronger association of cavitation with TB in our cohort may also partly reflect the radiological phenotype of diabetic TB rather than TB more broadly. Although all included patients underwent CT within 30 days of symptom onset, we did not stratify the analysis further by duration of illness as this would have reduced subgroup sizes and made interpretation difficult; temporal variation in disease stage may still have influenced imaging appearance in individual patients. Although imaging findings were recorded by one radiologist and verified by a second radiologist, formal interreader agreement was not assessed. Finally, some findings, including septic emboli, Rasmussen aneurysm, and empyema, were rare, limiting the precision with which their discriminatory value could be evaluated.

## CONCLUSION

Pulmonary melioidosis and pulmonary TB demonstrate substantial overlap on chest CT in diabetic patients, with no single imaging feature sufficiently specific to distinguish one from the other in isolation. Although cavitation favored TB in multivariable analysis, it was present in both diseases and cannot serve as a stand-alone discriminator. In TB-endemic regions where melioidosis remains underrecognized, awareness of this radiological overlap is essential to avoid diagnostic anchoring toward TB and to ensure timely initiation of appropriate investigations and therapy. Chest CT should, therefore, be interpreted as an adjunct to rather than a substitute for targeted microbiological diagnosis.

## Supplemental Materials

10.4269/ajtmh.26-0337 Supplemental Materials
