# Supplementary material for: Computed Tomographic Features Associated with Pulmonary Melioidosis versus Pulmonary Tuberculosis in Diabetic Patients: A Retrospective Comparative Cohort Study
Source: Am J Trop Med Hyg. 2026 Oct 13:tpmd260337. Online ahead of print. doi: 10.4269/ajtmh.26-0337 (PMC13675693; doi:10.4269/ajtmh.26-0337)
Supplement: Supplementary file 1 [file tpmd260337.SD1.pdf]

Supplementary Figure 1: Flow chart of patient selection. Among patients with microbiologically confirmed pulmonary tuberculosis and pulmonary melioidosis identified during the study period, those with available chest computed tomography performed within 30 days of symptom onset were screened for eligibility. Patients with documented diabetes mellitus and chest CT were included in the final analysis, yielding 105 patients with pulmonary tuberculosis and 46 with pulmonary melioidosis.

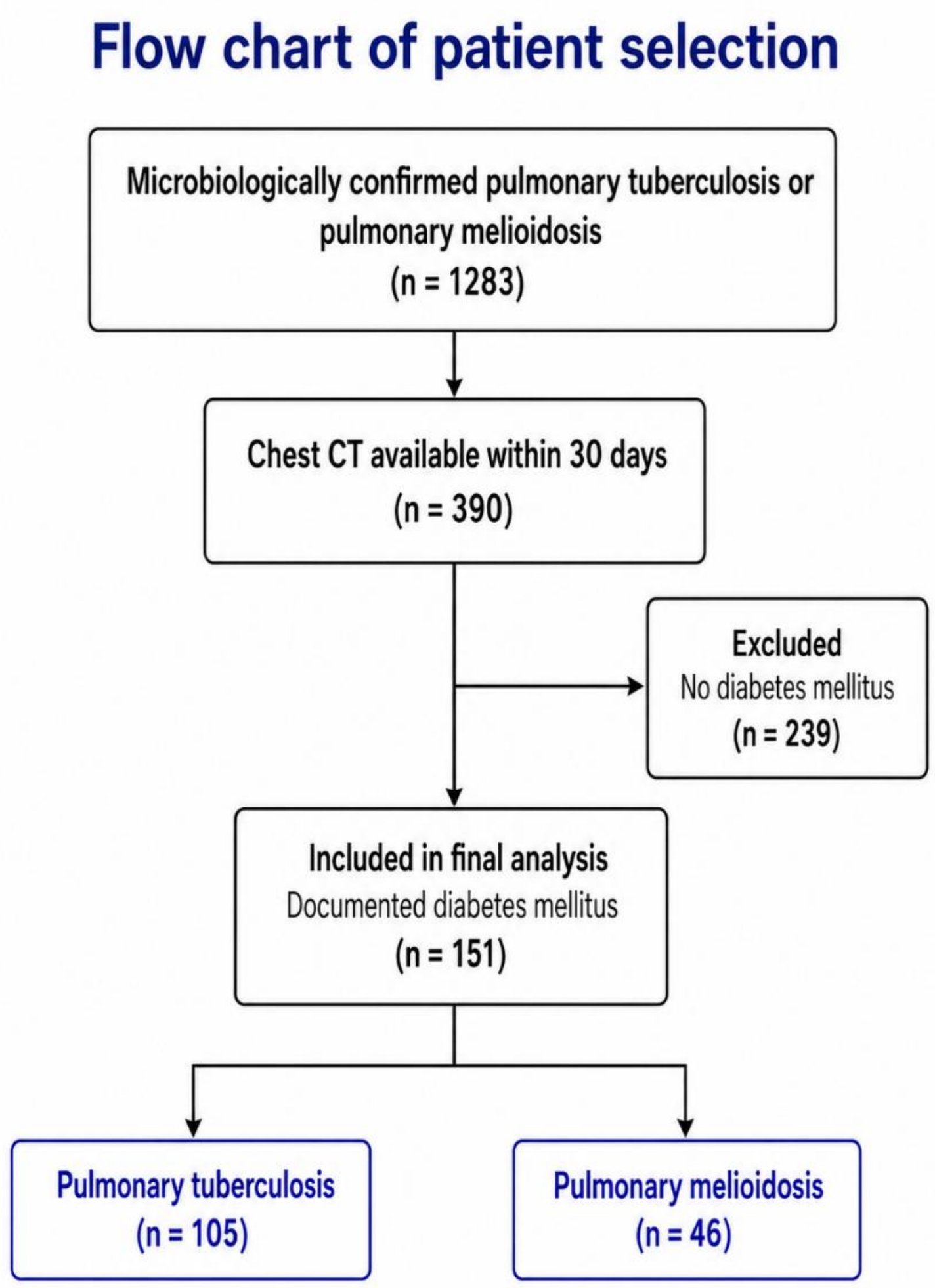

Supplementary File: STROBE checklist for observational studies

Section / Item	Recommendation	Page and line number(s) in final revised manuscript	Status
**1(a) Title**	Indicate the study design with a commonly used term in the title	p.1, lines 1–3	Reported
**1(b) Abstract**	Provide an informative and balanced summary of what was done and what was found	pp.1–2, Abstract section	Reported
**2 Background/rationale**	Explain the scientific background and rationale	p.3, Introduction, para 1–2	Reported
**3 Objectives**	State specific objectives, including any prespecified hypotheses	p.3, Introduction, final sentences	Reported
**4 Study design**	Present key elements of study design early in the paper	p.3, Methodology, first paragraph	Reported
**5 Setting**	Describe setting, location, and relevant dates	p.3, Methodology, first paragraph	Reported
**6(a) Participants**	Give eligibility criteria and sources/methods of selection of participants	p.3, Methodology, first paragraph	Reported
**6(b) Matched studies**	Give matching criteria and number of exposed and unexposed	Not applicable	N/A
**7 Variables**	Clearly define outcomes, exposures, predictors, potential confounders, and effect modifiers; give diagnostic criteria	pp.3–4, Methodology; Table 1; Table 2	Reported
**8 Data sources / measurement**	For each variable of interest, give sources of data and details of methods of assessment	pp.3–4, Methodology	Reported
**9 Bias**	Describe efforts to address potential sources of bias	p.3, Methodology, first paragraph; p.8, Limitations	Reported
**10 Study size**	Explain how the study size was arrived at	p.3, Methodology, second paragraph	Reported
**11 Quantitative variables**	Explain how quantitative variables were handled in the analyses	p.4, Methodology, paragraphs 3–4	Reported
**12(a) Statistical methods**	Describe all statistical methods, including those used to control for confounding	p.4, Methodology, statistical analysis paragraphs	Reported
**12(b) Subgroups / interactions**	Describe any methods used to examine subgroups and interactions	p.4, final sentences of Methodology	Reported as none performed
**12(c) Missing data**	Explain how missing data were addressed	p.4, final sentences of Methodology	Reported
**12(d) Loss to follow-up**	If applicable, explain how loss to follow-up was addressed	Not applicable	N/A
**12(e) Sensitivity analyses**	Describe any sensitivity analyses	p.4, final sentences of Methodology	Reported as none performed
**13(a) Participants**	Report numbers of individuals at each stage of study	p.4, Results, first sentence	Reported

**13(b) Non-participation**	Give reasons for non-participation at each stage	Added as Supplementary Figure 1	Reported
**13(c) Flow diagram**	Consider use of a flow diagram	Added as Supplementary Figure 1	Reported
**14(a) Descriptive data**	Give characteristics of study participants and information on exposures and potential confounders	p.4, Results, first paragraph; Table 2	Reported
**14(b) Missing data**	Indicate number of participants with missing data for each variable of interest	p.4, Methodology, final sentences	Partially reported
**14(c) Follow-up time**	Summarise follow-up time	Not applicable	N/A
**15 Outcome data**	Report numbers of outcome events or summary measures over time	pp.4–5, Results; Tables 3–4	Reported
**16(a) Main results**	Give unadjusted estimates and, if applicable, confounder-adjusted estimates and their precision	p.5, Results; Table 4	Reported
**16(b) Category boundaries**	Report category boundaries when continuous variables were categorised	p.4, Methodology; Table 1; Table 2	Reported
**16(c) Absolute risk**	If relevant, consider translating estimates of relative risk into absolute risk	Not applicable	N/A
**17 Other analyses**	Report other analyses done, such as subgroup or sensitivity analyses	p.4, Methodology	Reported as none performed
**18 Key results**	Summarise key results with reference to study objectives	pp.5–8, Discussion; especially opening and conclusion	Reported
**19 Limitations**	Discuss limitations of the study, taking into account sources of potential bias or imprecision	p.8, Limitations paragraph	Reported
**20 Interpretation**	Give a cautious overall interpretation considering objectives, limitations, multiplicity of analyses, results from similar studies, and other evidence	pp.5–8, Discussion throughout	Reported
**21 Generalisability**	Discuss the generalisability of the study results	p.8, Limitations and Conclusion	Reported
**22 Funding**	Give the source of funding and role of the funders	p.8, end matter, “Funding”	Reported
